# HLA DR phenotypic frequencies and genetic risk of Type 1 diabetes in west region of Algeria, Tlemcen

**DOI:** 10.1186/1471-2156-5-24

**Published:** 2004-08-24

**Authors:** Mourad Aribi, Soraya Moulessehoul, Ahmed-Bakir Benabadji, Mohammed Kendoucitani

**Affiliations:** 1 Department of Biology, Djillali Liabes University, Sidi-Bel-Abbès, 22 000, Algeria; 2 Department of Pharmacy, Abou-Bekr Belakaïd University, Tlemcen, 13 000, Algeria; 3 Department of Medicine, Internal Medicine Board, Medical Centre University of Tlemcen, 13 000, Algeria

## Abstract

**Background:**

The main genomic region controlling the predisposition to type 1 diabetes is the Human Leukocyte Antigens (HLA) class II of the major histocompatibility complex. Association with different HLA types depends also on the studied populations. In our investigation, we tried to measure the phenotypic HLA class II association frequencies of DR3 and/or DR4 antigens, using a serologic method called microlymphocytotoxicity analysis, in diabetic and nondiabetic (ND) subjects originating from the west-Algerian region of Tlemcen. The aim of the present study was to determine which HLA DR antigens represent a high susceptibility to develop the disease in this area. Using a case-control retrospective study design, we randomly recruited ninety-one related subjects, 39 type 1 diabetics and 52 ND as controls, at the Internal Medicine Board of Medical Centre University of Tlemcen.

**Results:**

DR3 antigen frequencies were comparable between the type 1 diabetics and the ND subjects and showed no association with the disease (*p *= 1.000, OR = 0.95), whereas DR4 and DR3DR4 antigens were associated with susceptibility to develop type 1 diabetes (DR4; OR = 2.10, DR3DR4; OR = 1.30). Also, no incidence for DR3 (*p *= 0.2646) or DR3DR4 (*p *= 0.0699) antigen frequencies was related to the sex ratio. However, significant differences in HLA DR4 frequencies between type 1 diabetics and ND were found to be related to sex (*p *= 0.0085).

**Conclusion:**

Taken together, our investigation showed that the strongest association with type 1 diabetes was noticed in the presence of HLA DR4 antigens followed by DR3DR4 antigens. This study highlighted a characteristic of Tlemcen population; a history of consanguineous marriages. Association studies between the disease and genetic polymorphisms should be undertaken in a population where consanguinity is more limited to reduce confounding in result interpretations.

## Background

The type 1 diabetes, previously called insulin-dependent diabetes mellitus (IDDM), is the consequence of progressive and selective destruction of pancreatic β cells by an immune-mediated process [1], resulting in an absolute lack of insulin [2]. It is well established that this self-destruction is primarily provoked by the activation of the autoreactive T lymphocytes by the production of T-helper 1 cytokines [3,4]. In addition, the difference of the epidemiological data from one region to another could largely explain why the release of the autoimmunity is stimulated under the influence of one or more environmental factors [5,6], in genetically predisposed subjects. It is currently obvious that the strongest genetic susceptibility of predisposition is allotted to the IDDM1 alleles located in the HLA locus of the chromosome 6p21 [7-9], and the non-HLA alleles, particularly the IDDM2 polymorph gene located in the region 5' of the insulin gene (*INS*) promoter situated on the chromosome 11p15 [10,11]. Other regions of the genome were identified as IDDM3, coding for the IGF1 receptor, IDDM4, located near the fibroblast growth factor 3 gene, IDDM5, near the estrogens receptor gene [7] etc. The majority of these regions have no possible statistical criteria allowing them to be clearly linked to the disease [11]. In all cases, it is mainly HLA alleles that present a high risk of contracting the disease compared to non-HLA alleles [6,12]. Due to this, they are qualified as high genetic predictive markers of developing type 1 diabetes in families having a type 1 diabetic member. Interestingly, the disease prediction offered with success the possibility of clinical trials to delay, or even prevent the appearance of type 1 diabetes.

However, the different HLA types associated with diabetes depend also on the population. The purpose of our study is to measure HLA DR3 and/or DR4 antigen frequencies and their association in diabetic and nondiabetic subjects originating from the west-Algerian region of Tlemcen. Using a case-control retrospective study design, we attempt to determine which is the greatest HLA DR susceptibility contributing to developing type 1 diabetes. Ninety-one (91) eligible subjects (thirty-nine (39) type 1 diabetics and fifty-two (52) nondiabetics with relatives with type 1 diabetes as controls), were recruited at the Internal Medicine Board of the Medical Centre University of Tlemcen.

## Results

Table 1 summarizes HLA DR3, DR4 and DR3DR4 antigen frequencies in type 1 diabetics and their nondiabetic relatives.

**Table 1:** HLA DR phenotypic frequencies according to *p*-values and Odds ratio in type 1 diabetic and nondiabetic subjects.

	**Type 1 diabetics n = 39: 15 M/24 F**		**Nondiabetic controls n = 52: 21 M/31F**			
			
	**Frequency (Proportion and %)**					
		
**HLA**	**M**	**F**	**M**	**F**	***p***	**Odds ratio (95 % CI)**
**DR3**	3 (7.69)	7 (17.9)	5 (9.62)	9 (17.31)	0.2646^a^	0.95 (0.48–1.87)
	10 (25.64)		14 (26.92)		1.000	
**DR4**	4 (10.26)	9 (23.08)	6 (11.54)	4 (7.69)	0.0085 ^a, ^*	2.10 (1.04–4.24)
	13 (33.33)		10 (19.23)		0.0361*	
**DR3DR4**	4 (10.26)	4 (10.26)	2 (3.85)	7 (13.46)	0.0699^a^	1.30 (0.60–2.80)
	8 (20.51)		9 (17.31)		0.5887	
**DR3 and/or DR4**	11 (28.21)	20 (51.28)	13 (25.0)	20 (38.46)	0.5791^a^	2.21 (1.13–4.36)
	31 (79.49)		33 (63.46)		0.0194*	
**X/X**	4 (10.26)	4 (10.26)	8 (15.38)	11 (21.15)	0.0111 ^a, ^*	-
	8 (20.51)		19 (36.54)		0.0194*	

^a^*p*-Values related to the sex ratio comparison (male = M, female = F). * *p *< 0.05. X = non-DR3, non-DR4.

**Figure 1:**
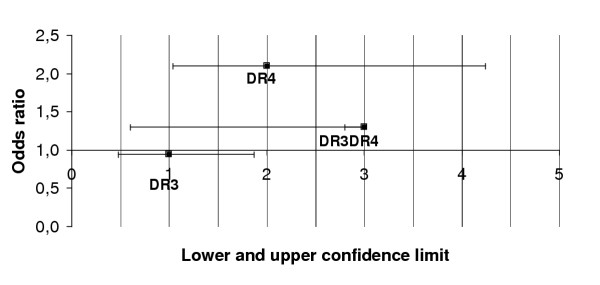
**HLA DR antigen associations with type 1 diabetes. **The block boxes represent the odds ratios (OR). The horizontal lines represent the lower and upper confidence limit of OR (confidence interval, 95 % CI).

DR4 and DR3DR4 antigens showed an association with susceptibility to type 1 diabetes (DR4; OR = 2.10, DR3DR4; OR = 1.30, that is respectively OR confidence interval 1.04–4.24 and 0.60–2.80, 95% CI), in contrast, DR3 antigens showed no association with the disease (OR = 0.95, OR confidence interval 0.48–1.87, 95% CI). It is important to notice that the strongest association is found in DR4 phenotype as indicated in Figure 1.

**Figure 2:**
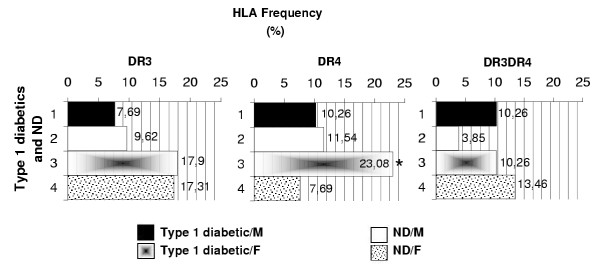
HLA DR antigen frequencies compared to the sex ratio. * *p *< 0.05, Type 1 diabetics vs. control subjects.

In addition, the phenotypic frequency of DR4 or DR3DR4 molecules is higher in diabetic group than in the control one, although the difference did not reach significance level in DR3DR4 frequencies (*p *= 0.0361 and *p *= 0.5887 respectively). On the contrary, the DR3 molecules frequency is slightly decreased in type 1 diabetic patients compared to the controls and presents no statistically significant difference (*p *= 1.000). Furthermore, no incidence was related to the sex criteria for the frequencies of DR3 and DR3DR4 molecules (*p *> 0.05). However, significant differences in HLA DR4 frequencies are linked to the female sex and present a value definitely higher in type 1 diabetic patients compared to those of nondiabetics for the same sex (*p *< 0.05) (Figure 2).

## Discussion

Type 1 diabetes is a polygenic disease which results from the interaction between environmental (viral, toxic, nutritional, socioeconomic [6]) and genetic factors. It is the form of diabetes which occurs mainly in children and young adults [13].

Fortunately, molecular epidemiology offers the hope of the possibility of preventing the disease in the future, by evaluating the potential factors of risk of developing this pathology [6]. Although almost 90 % of new cases of type 1 diabetes occur sporadically, studies of individuals with a diabetic relative in their family are essential [4].

It is well established that associations between type 1 diabetes and certain HLA antigens largely facilitate the identification of the subjects having a potential risk to develop the disease. Among the putative HLA molecules known to confer a high susceptibility are DQA1 (*0301)-DQB1 (*0302) (= DQ8), DQA1 (*0501)-DQB1 (*0201) (= DQ2), DRA-DRB1 (*0401) (= DR4) and DRA-DRB1 (*0301) (= DR3) [14]. However, epidemiological studies showed that the different HLA types associated with the diabetes depend also on the various populations. For instance, the risk of developing type 1 diabetes in Caucasians is greater if they are carrying the HLA A8 and B15, while DQ6 alleles are protectors [11,14,15]. Among Japanese, it is the association with the HLA B54 that confers a higher susceptibility to develop the disease, while the strongest association was found with HLA DR and HLA DQ locus [11].

In Algeria, and especially in its western region (Tlemcen), which is known for its history of consanguineous marriage, there is a high rate of consanguinity. This fact could increase the risk of developing type 1 diabetes by favouring the transmission of HLA haplotypes and recessive genes of susceptibility except for HLA antigens that are common in both parents. For these reasons, we were interested in checking whether phenotypically DR3DR4 antigens would involve less risk to develop the disease, in comparison with DR3 or DR4 antigens, knowing the fact that the presence of a probable consanguinity could reduce the frequency of DR3DR4 polymorph phenotype compared to that of DR3 or DR4 phenotype.

On a purely comparative basis, similarities seem to be found between our results and those of other investigators [5,13,15] with regards to HLA DR4 or DR3DR4 frequencies, which are higher in the type 1 diabetic than the nondiabetic population. On the contrary, the HLA DR3 antigens showed comparable frequencies in both groups of our sample. Consequently, these observations associating DR3 phenotype to a protector effect against type 1 diabetes in our studied population are thus do not conform with those reported in the literature [15-18]. However, DR4 and DR3DR4 antigens are obviously associated with susceptibility of developing the disease. It should also be noted that DR3DR4 antigens might represent a weaker predictive value of disease risk. We concluded from this that the non-excess of DR3DR4 antigens, or comparable frequencies of DR3 molecules between type 1 diabetic patients and ND relative controls, might be a strong indices of consanguinity in our sample. A recent study carried out in Sweden showed that HLA DR3 is associated to the development of type 1 diabetes and the incompatibility of blood group ABo [19]. One can thus note that the role of the HLA DR3 antigens in conferring risk for type 1 diabetes can be masked in the homogeneous populations. Due to the restricted size of our sample, which may influence our interpretation, we should not consider the odds ratio of HLA DR3 antigens or the eventual consanguinity of our studied population. Moreover, many evidences [20-23] incriminate DR3 and/or DR4 antigens in the susceptibility to type 1 diabetes and their association with the detected autoantibodies in this disease. Indeed, the marks of autoimmunity are much more observed in the diabetic patients carrying DR3 or DR4 antigens than in the diabetic patients where DR3 or DR4 antigens were absents [13]. Furthermore, recent research indicates that a subject with HLA DR4 or DR3 alleles has three or four times more chance of developing type 1 diabetes compared to the general population; DR3DR4 is associated with the highest risk (20 to 40 times more) [11].

In Algeria, very few investigations have been undertaken to study the impact of the genetic background on the risk to develop type 1 diabetes in its population, where an annual average incidence of 4.7 per 100000 has already been listed [24]. In a similar study carried out on Algerian unrelated type 1 diabetics (n = 50) and nondiabetics controls (n = 46), the presence of DR3-DQ2 (linkage disequilibrium) in 45 % of patients and in 13 % of controls was detected by molecular genotyping method using PCR (polymerase chain reaction) and SSO (sequence specific oligonucleotide). DR4-DQ8 was found in 37 % of diabetic cases and in 4 % of control groups [25]. Finally, association with type 1 diabetes attributed to DR-B1*0405 (alleles of DR4 antigens expression) susceptibility showed a match with our results; however, the DR4 antigens were found to be linked to the female sex. According to the results showed in Table 1, there is no difference between men and women patients with type 1 diabetes carrying DR4 antigens, but a significant difference was noticed in female sex, either women were contracted or not with diabetic disease.

It is certain that screening of HLA class II sub-types and determination of DNA coding sequences allows more precise characterization of ethnic groups, especially, because the same coded molecules can differ by the position of few amino acids. For example, it is well established that DQA1 and DQB1 alleles (sub-types of HLA DQ) code respectively for the alpha and beta chain of the DQ molecule [6]. Thus, the combination of DQ alpha with Arg in position 52 (Arg-52) and DQ beta in position 57 without Asp (non-Asp 57) is called a diabetogenic heterodimer which is the biggest risk factor of type 1 diabetes in Caucasian. Nevertheless, among the Japanese population, type 1 diabetes is particularly associated with HLA DQ alpha Arg-52, but not with HLA DQ beta non-Asp 57 [5].

Moreover, it is true that the search of an association with a candidate gene allows a better characterization for most of the frequent multifactorial diseases, because the candidate genes are directly implied in the pathological processes. Today, it is reported that the CTLA-4 (cytotoxic T-lymphocyte antigen-4) allele, localised on the chromosome 2p33, former IDDM12, is largely associated with the susceptibility of numerous common complex diseases, such as the common autoimmune disorders Graves' disease, the autoimmune hypothyroidism and type 1 diabetes [26]. Combined, these pertinent observations still open new perspectives for debating this crucial subject concerning the public health.

## Conclusion

Type 1 diabetes, or youth diabetes, is a multifactorial disease occurring on a genetic ground of predisposition and starts under certain environmental conditions. The most effective preventive strategy must be designed at the pre-diabetes stage, since immune and/or genetic markers can easily indicate subjects with high risk before the clinical symptoms. Thus, the genetic analysis of HLA class II associations has allowed a screening of the contributing molecules to the type 1 diabetes development and the selection of subjects, which are likely to contract the disease.

In this study, we were confronted with difficulties in interpretation of our results which are mainly due to the presence of some indices of consanguinity in our studied population, showing a non excess of DR3DR4 phenotype compared to the DR3 or DR4 phenotype, and a no link of DR3 phenotype to the disease. This result could be due to an ethnic characteristic of Tlemcen population, a history of consanguineous marriages. Nevertheless, these preliminary results made it possible to answer the asked questions. Thus, the strongest type 1 diabetes association is statistically revealed with phenotypic expression of DR4 followed by DR3DR4 phenotype. Association studies between the disease and genetic polymorphisms should be carried out on the population having more limited consanguinity to reduce confusions in result interpretations.

## Methods

### Subjects

The study was conducted on ninety-one first-degree related subjects (brothers, sisters and siblings), randomly recruited at the Internal Medicine Board of the Medical Centre University of Tlemcen (west-Algeria). The sample included thirty-nine patients with type 1 diabetes (15 males, 24 females), and fifty-two healthy subjects (21 males, 31 females) selected from diabetes relatives as controls. Prior medical histories and personal characteristics were obtained from participants *via *a questionnaire. The patients' mean (± Standard Deviation) age at clinical onset was 12.28 ± 5.97 years with range of 5 to 22 years and median of 11 years. Subjects who were not first degree related and who were not originating from Tlemcen region were excluded. The use of first-degree relatives eliminates exposures of environmental factors such as food items and viruses since first-degree relatives usually share the same milieu. For execution of the protocol, the informed consent was obtained from all the participating subjects to the designated study.

### HLA phenotyping (standard complement-dependent assay)

The applied serologic technique lies on the aptitude of the antibodies to recognize allotropic determinants of HLA molecules on cellular surface [27]. This method is sensitized by a reaction of microlymphocytotoxicity [28,29], which uses specific anti-HLA DR antisera and rabbit complement of commercial typing tray (Biotest, Germany). Initially, the peripheral blood lymphocytes (PBL) were separated from the other illustrated elements of venous blood (collected in EDTA-containing tubes) by density gradient centrifugation on Ficoll-Hypaque [30]. B lymphocytes were isolated by using nylon-wool-separated columns [31].

### Statistical analysis

The comparison of phenotypic frequencies was obtained by using chi-square analysis with Yates' correction or by Fisher's Exact test, whenever appropriate. The application of the observed χ^2 ^vs. expected χ^2 ^was employed to show significance of frequency differences with the sex ratio. A *p *value of less than 0.05 was considered statistically significant (two-by-two table: degrees of freedom (df) = 1, chi-square ≥ 3.84) [32]. The association between HLA antigens and type 1 diabetes was performed by determination of odds ratio (OR) [33,34] (confidence interval, 95 % CI). All statistical analyses were performed using the Epi Info 2000 Version 1.0 for Windows 95, 98, NT, and 2000 computers (Epi Info, Atlanta, Georgia, USA) and STATISTICA Version 5.0, '97 (STATISTICA, StatSoft, Paris, France).

## Author's contributions

AM drafted the manuscript, performed statistical analyses and carried out the bibliography research. MS participated by coordinating and orienting the designated study. BA carried out HLA phenotyping and participated in the study design. KM recruited the eligible subjects. All authors read and approved the final version of the manuscript.
